# Supplementary material for: A Narrative Review of Health Status and Healthcare Delivery in the Oil and Gas Industry: Impacts on Employees, Employers, and Local Communities
Source: Healthcare (Basel). 2023 Nov 2;11(21):2888. doi: 10.3390/healthcare11212888 (PMC10647413; doi:10.3390/healthcare11212888)
Supplement: Supplementary file 1 [file healthcare-11-02888-s001.zip › healthcare-2650050-supplementary.pdf]

Supplementary Table S1. Complete search strategy for each database

Search topic	Database	Search strategy	Number of results
Health of employees working in the mining industry (N = 1,239)	PubMed	((((((miner[Title/Abstract]) OR (miners[Title/Abstract]) OR (mining industry[Title/Abstract])) OR (resource industry[Title/Abstract])) OR (resource sector[Title/Abstract]))) NOT (data mining[Title/Abstract])<br><br>AND<br><br>((((((((((((((physical health[MeSH Terms]) OR (health behavior*[MeSH Terms])) OR (sleep[MeSH Terms])) OR (stress[MeSH Terms])) OR (mental health[MeSH Terms])) OR (fatigue[MeSH Terms])) OR (alcohol[Title/Abstract])) OR (smoking[Title/Abstract])) OR (diet[Title/Abstract])) OR (exercise[Title/Abstract])) OR (distress[MeSH Terms])) OR (sick*[MeSH Terms])) OR (ill*[MeSH Terms])) OR (wellbeing[MeSH Terms]))	241
	EMBASE	('miner'/de OR 'mining'/de OR 'resource industry':ti,ab,kw OR 'resource sector':ti,ab,kw) NOT 'data mining'/de<br><br>AND<br><br>'health'/de OR 'health behavior'/de OR 'sleep'/de OR 'stress':ti,ab,kw OR 'mental health'/de OR 'fatigue'/de OR 'alcohol'/de OR 'smoking'/de OR 'diet'/de OR 'exercise'/de OR 'distress':ti,ab,kw OR sick* OR ill* OR 'wellbeing'/de	720
	CINAHL and PsycINFO	TI miner OR AB miner OR TI miners OR AB miners OR TI “mining industry” OR AB “mining industry” OR TI “resource industry” OR AB “resource industry” OR TI “resource sector” OR AB “resource sector” NOT TX “data mining”<br><br>AND<br><br>“physical health” OR “health behavior” OR “health behaviour” OR sleep OR stress OR “mental health” OR fatigue OR TI alcohol OR AB alcohol OR TI smoking OR AB smoking OR TI diet OR AB diet OR TI exercise OR AB exercise OR distress OR sick* OR ill* OR wellbeing	278
Health status of remote and FIFO workers (N = 78)	PubMed	(((((((FIFO[Title/Abstract]) OR (fly-in-fly-out[Title/Abstract])) OR (fly in fly out[Title/Abstract])) OR (DIDO[Title/Abstract])) OR (drive-in drive-out[Title/Abstract])) OR (drive in drive out[Title/Abstract])) OR (long distance commut*[Title/Abstract]))<br><br>AND<br><br>((((((((((((((physical health[MeSH Terms]) OR (health behavior*[MeSH Terms])) OR (sleep[MeSH Terms])) OR (stress[MeSH Terms])) OR (mental health[MeSH Terms]))	21

		OR (fatigue[MeSH Terms])) OR (alcohol[Title/Abstract])) OR (smoking[Title/Abstract])) OR (diet[Title/Abstract])) OR (exercise[Title/Abstract])) OR (distress[MeSH Terms])) OR (sick*[MeSH Terms])) OR (ill*[MeSH Terms])) OR (wellbeing[MeSH Terms]))	
	EMBASE	'health'/de OR 'health behavior'/de OR 'sleep'/de OR 'stress':ti,ab,kw OR 'mental health'/de OR 'fatigue'/de OR 'alcohol'/de OR 'smoking'/de OR 'diet'/de OR 'exercise'/de OR 'distress':ti,ab,kw OR sick* OR ill* OR 'wellbeing'/de<br><br>AND<br><br>'fifo':ti,ab,kw OR 'fly-in fly-out':ti,ab,kw OR 'fly in fly out':ti,ab,kw OR 'dido':ti,ab,kw OR 'drive-in drive-out':ti,ab,kw OR 'drive in drive out':ti,ab,kw OR 'long distance commut*':ti,kw	23
	CINAHL and PsycINFO	“physical health” OR “health behavior” OR “health behaviour” OR sleep OR stress OR “mental health” OR fatigue OR TI alcohol OR AB alcohol OR TI smoking OR AB smoking OR TI diet OR AB diet OR TI exercise OR AB exercise OR distress OR sick* OR ill* OR wellbeing<br><br>AND<br><br>TI FIFO OR AB FIFO OR TI “fly-in fly-out” OR AB “fly-in fly-out” OR TI “fly in fly out” OR AB “fly in fly out” OR TI DIDO OR AB DIDO OR TI “drive-in drive-out” OR AB “drive-in drive-out” OR TI “drive in drive out” OR AB “drive in drive out” OR TI “long distance commut*” OR AB “long distance commut*”	34
Healthcare in mining communities (N = 659)	PubMed	((mining communit*[Title/Abstract]) OR (mining town*[Title/Abstract])<br><br>AND<br><br>((((((healthcare[MeSH Terms]) OR (health care[MeSH Terms])) OR (models of care[MeSH Terms])) OR (health service[MeSH Terms])))	19
	EMBASE	(mining AND communit*:ti,ab,kw) OR (mining AND town*:ti,ab,kw)<br><br>AND<br><br>'health care'/de OR 'health service'/de OR 'models of care':ti,ab,kw	16
	CINAHL and PsycINFO	TI “mining communit*” OR AB “mining communit*” OR TI “mining town*” OR AB “mining town*”<br><br>AND<br><br>healthcare OR “health care” OR “health service*” OR “models of care”	11
	Scopus	(TITLE-ABS (mining AND communit*) OR TITLE-ABS (mining AND town*))	613

		AND<br><br>((TITLE-ABS (healthcare) OR TITLE-ABS (health AND care) OR TITLE-ABS (health AND service*) OR TITLE-ABS (models AND of AND care)))	
Healthcare in remote communities (N = 6,155)	PubMed	(((remote communit*[Title/Abstract]) OR (isolated communit*[Title/Abstract])))<br><br>AND<br><br>((((((healthcare[MeSH Terms]) OR (health care[MeSH Terms])) OR (models of care[MeSH Terms])) OR (health service[MeSH Terms])))	573
	EMBASE	(isolated AND communit*:ti,ab,kw) OR (remote AND communit*:ti,ab,kw)<br><br>AND<br><br>'health care'/de OR 'health service'/de OR 'models of care':ti,ab,kw	337
	CINAHL and PsycINFO	"remote communit*" OR AB "remote communit*" OR TI "isolated communit*" OR AB "isolated communit*"<br><br>AND<br><br>healthcare OR "health care" OR "health service*" OR "models of care"	485
	Scopus	(TITLE-ABS (healthcare) OR TITLE-ABS (health AND care) OR TITLE-ABS (health AND service*) OR TITLE-ABS (models AND of AND care))<br><br>AND<br><br>(TITLE-ABS (remote AND communit*) OR TITLE-ABS (isolated AND communit*))	4760
Trauma and emergency response in remote and mining communities (N = 2,041)	PubMed	((((trauma[MeSH Terms]) OR (emergency response[MeSH Terms])) OR (major injur*[MeSH Terms])) OR (advanced life support[MeSH Terms])<br><br>AND<br><br>(((remote[Title/Abstract]) OR (isolated communit*[Title/Abstract]) OR (mining communit*[Title/Abstract) OR (mining town*[Title/Abstract)))	1,152
	EMBASE	'remote communit*':ti,ab,kw OR 'isolated communit*':ti,ab,kw OR 'mining communit*':ti,ab,kw OR 'mining town*':ti,ab,kw<br><br>AND	37

		‘injury'/exp OR 'injury' OR 'emergency response'/exp OR 'emergency response' OR 'major injur*':ti,ab,kw OR 'advanced life support'/exp OR 'advanced life support'	
	CINAHL and PsycINFO	trauma OR “emergency response” OR “major injur*” OR “advanced life support”<br><br>AND<br><br>TI “remote communit*” OR AB “remote communit*” OR TI “isolated communit*” OR AB “isolated communit*” OR TI “mining communit*” OR AB “mining communit*” OR TI “mining town*” OR AB “mining town*”	28
	Scopus	(TITLE-ABS-KEY (remote AND communit*) OR TITLE-ABS-KEY (isolated AND communit*) OR TITLE-ABS-KEY (mining AND communit*) OR TITLE-ABS-KEY (mining AND town*))<br><br>AND<br><br>(TITLE-ABS-KEY (trauma) OR TITLE-ABS-KEY (emergency AND response) OR TITLE-ABS-KEY (major AND injur*) OR TITLE-ABS-KEY (advanced AND life AND support))	824
Healthcare in the oil and gas industry (N = 4,613)	PubMed	((((oil and gas[Title/Abstract]) OR (energy sector[Title/Abstract])) OR (energy industr*[Title/Abstract]))<br><br>AND<br><br>((((((((healthcare[MeSH Terms]) OR (health care[MeSH Terms])) OR (health service*[MeSH Terms])) OR (models of care[MeSH Terms])) OR (trauma[MeSH Terms])) OR (emergency response[Title/Abstract])) OR (telehealth[MeSH Terms])) OR (telemedicine[MeSH Terms]))	195
	EMBASE	'oil and gas':ti,ab,kw OR 'energy sector':ti,ab,kw OR 'energy industr*':ti,ab,kw<br><br>AND<br><br>'health care'/de OR 'health service'/de OR 'models of care':ti,ab,kw OR 'injury'/de OR 'emergency response'/de OR 'telehealth'/de OR 'telemedicine'/de	17
	CINAHL and PsycINFO	TI (oil and gas) OR AB (oil and gas) OR TI energy sector OR AB energy sector OR TI energy industr* OR AB energy industr*<br><br>AND<br><br>healthcare OR health care OR health service* OR models of care OR trauma OR telehealth OR telemedicine OR TI emergency response OR AB emergency response	112
	Scopus	#1 oil and gas, energy sector, energy industr*<br>AND	4,289

		#2 healthcare, health care, health service*, models of care, trauma, emergency response, telehealth, telemedicine	
	Google Scholar	oil AND gas AND healthcare	NA
Public-private partnerships in healthcare	Google	#1 "public private partnership" AND Health AND Australia<br><br>#2 Partnership AND health AND (oil OR gas)	NA
	Google Scholar	#1 "public private partnership" AND Health AND Australia<br><br>#2 Partnership AND health AND (oil OR gas)	NA

Note: NA = Not applicable, CINAHL and PsycINFO were both searched via EBSCO host and therefore, the search strategy and number of results are combined, all database searches were restricted to articles published in English within the preceding 10 years (2013-2023), number of results not reported for grey literature as only the first 50 pages of results were screened.

Supplementary Table S2. Key findings from relevant articles retrieved via the literature search

Health status and safety of employees in the resource sector		
**Author, year**	**Title**	**Key findings**
Alroomi & Mohamed, 2021 [1]	Occupational stressors and safety behaviour among oil and gas workers in Kuwait: The mediating role of mental health and fatigue	• Stress in workers results in them acting less safely at work, which is mediated by mental health and fatigue.
Bauerle, Dugdale & Poplin, 2018 [2]	Mineworker fatigue: A review of what we know and future decisions	• Miners are exposed to specific health risks due to the characteristics of their work and work environment, namely environmental stressors, shift work, long working hours, FIFO/DIDO models, and sleep disruptions (e.g., sleeping in unfamiliar environments).<br>• As a consequence of these factors, fatigue can manifest as physical, physiological, cognitive, and behavioural concerns which can lead to other health consequences.
Benson et al., 2021 [3]	Assessing the common occupational health hazards and their health risks among oil and gas workers	• Health hazards to workers in the oil and gas industry encompasses chemical hazards, physical hazards, psychosocial hazards, ergonomic hazards, and biological hazards.<br>• Risk management and supervision should be implemented in the workplace and compulsory medical testing should be carried out to monitor the health status of workers.
Bresic et al., 2007 [4]	Stress and work ability in oil industry workers	• Regardless of the nature of the work (e.g., office/laboratory/field), oil company workers reported being exposed to stress.
Carrington & McIntosh, 2013 [5]	A literature review of wellness, wellbeing and quality of life issues as they impact upon the Australian mining sector	• Workers in the mining industry have low levels of work-life balance.<br>• Mine workers often get limited input into shifts/hours worked.<br>• Work-life balance and quality of life are the most important considerations of mine workers when they decide whether to accept a work offer and accommodation arrangements.<br>• FIFO/DIDO models can be detrimental to mine worker wellbeing.<br>• Mine workers (especially FIFO/DIDO) are more likely to smoke, have higher levels of alcohol consumption and be physically inactive compared to the general population.<br>• FIFO/DIDO workers are at a high risk of alcohol/tobacco/drug use, poor diet, physical inactivity, sexually transmitted/blood borne infections, mental health issues, and fatigue-related injury.<br>• Substantial costs associated with absenteeism, poor health, and staff turnover are also documented.
Considine et al., 2017 [6]	The contribution of individual, social and work characteristics to employee mental health in a coal mining industry population	• K10 scores were low (61%), moderate (26%), high (10%) and very high (3%), which after controlling for age and gender, were higher than national data (drawn from an Australian health survey; general population).<br>• Low SES had a minor contribution to distress and low social networks were associated with higher levels of distress.<br>• Distress was associated with previous history of depression, anxiety, and drug/alcohol problems.

		• Those in managerial positions reported higher levels of distress.<br>• Dissatisfaction with work, job insecurity, working in mining for financial reasons, and perceiving that the organisation was not committed to employee mental health were also associated with higher levels of distress.
Deng, He & Li, 2021 [7]	Factors influencing job burnout and musculoskeletal disorders among coal miners in the Xinjiang Uygur Autonomous region	• Job-related burnout was moderate in 45% male and 29% females and severe in 7% males and 3% females.<br>• Burnout was lowest in those on day shifts.
Gibson-Smith, 2016 [8]	Promoting and implementing self-care: A mixed methods study of offshore workers and remote healthcare practitioners	• Offshore workers scored poorly on multiple domains (BMI, diet, alcohol, insomnia, physical activity, and smoking).<br>• Oil and gas workers may benefit from lifestyle interventions relating to healthy eating, physical activity, and alcohol consumption.<br>• Remote healthcare practitioners reported that offshore workers diet and alcohol consumption were in particular need of behaviour change.
Gibson Smith et al., 2018 [9]	Health, self-care and the offshore workforce: Opportunities for behaviour change interventions, an epidemiological survey	• Over 70% were classified as overweight or obese and 15% reported a long-term illness, of which 80% were taking medication.<br>• Median SF-8 scores were 56.1 (physical) and 54.7 (mental), representing high physical and mental quality of life.<br>• Over 50% were at risk of harmful alcohol use and 67% had poor sleep quality.<br>• 55% of participants met the recommended fruit and vegetable intake per day and 70% met the recommended physical activity guidelines.
Hagan-Haynes et al., 2022 [10]	On the road again: A cross-sectional survey examining work schedules, commuting time, and driving-related outcomes among U.S. oil and gas extraction workers	• Long daily commutes, work schedules, sleep restriction on workdays, and lack of policies lead to riskier driving-related behaviours of workers.
James et al., 2018 [11]	Correlates of psychological distress among workers in the mining industry in remote Australia: Evidence from a multi-site cross-sectional study	• 44% reported moderate – very high levels of psychological distress (K10).<br>• Distress declined with age.<br>• A history of anxiety/depression/drug or alcohol problems we associated with psychological distress.<br>• Distress was higher in participants who had concerns of losing their job, worked rotating shifts, worked more than 12-hour shifts, or work in mining for 3-10 years.
James et al., 2021 [12]	Alcohol consumption in the Australian mining industry: The role of workplace, social, and individual factors	• Harmful alcohol use was associated with being younger, male, and exhibiting higher levels of psychological distress.
Kvalheim & Dahl, 2016 [13]	Safety compliance and safety climate: A repeated cross-sectional study in the oil and gas industry	• Safety climate was predictive of safety compliance in the oil and gas industry.

Le, Balogun & Smith, 2022 [14]	Long work hours, overtime, and worker health impairment: A cross-sectional study among stone, sand, and gravel mine workers	• Mean BMI was 30 ± 6 (overweight category).<br>• Long work hours (>60 hours per week) were associated with stress and burnout.
Liu et al., 2020 [15]	The state of occupational health and safety management frameworks (OHSMF) and occupational injuries and accidents in the Ghanian oil and gas industry: Assessing the mediating role of safety knowledge	• Safety training predicted safety knowledge, work-related injuries, and workplace accidents.
McLean, 2012 [16]	Mental health and well-being in resident mine workers: Out of the fly-in fly-out box	• Even for resident (non-FIFO) mine workers, the nature of the work can impact mental health and wellbeing.<br>• Personal and organisational support can positively impact mental health and wellbeing.<br>• Support related to relationships, lifestyle, work characteristics, and positive attitudes surrounding mental health. This included close working relationships with peers and management, as well as within the organisational structure.
McPhedran & De Leo, 2014 [17]	Relationship quality, work-family stress, and mental health among Australian male mining industry employees	• Employment in the mining industry was not associated with higher levels of work/family stress.
Miller et al., 2019 [18]	Suicide risk and social support in Australian resource sector employees: A cross-sectional study	• Employees in the resource sector reported high levels of psychological distress, including elevated suicide risk.<br>• No particular types of social support were superior in reducing distress.
Miller et al., 2020 [19]	Depression, suicide risk, and workplace bullying: A comparative study of fly-in, fly-out and residential resource workers in Australia	• One third of resource workers experience psychological distress (FIFO and residential).<br>• Suicide risk and depression were related to bullying and social support.
Moscicka-Teske et al., 2019 [20]	The relationship between psychosocial risks and occupational functioning among miners	• Less psychosocial risk in the workplace is related to increased job satisfaction and work commitment and reduced turnover intention.
Pavicic Aezelj et al., 2019 [21]	Anxiety and depression symptoms among gas and oil industry workers	• 15% of workers reported anxiety and depression symptoms.<br>• Young workers, those on longer rotations, and those with less service years reported more anxiety/depression symptoms.
Pelders & Nelson, 2019 [22]	Contributors to fatigue of mine workers in the South African gold and platinum sector	• Fatigue was related to being younger, low exercise, poor nutrition, less sleep, alcohol use, poor health status, more sick leave, higher stress, lower job satisfaction, extended working hours, and high workloads.
Rasmussen & Ahsan, 2022 [23]	The safety programme as a tool of improvement for safety culture in the workplace: An exploratory follow-up	• A new safety programme was introduced to all employees/contractors in 2010 and was divided into engagement, motivation, conversion and maintenance phases:

Author, year	Title	Key findings
	study from the Danish offshore oil and gas sector	▪ Engagement: Employees participated in workshops and some (32) in train the trainer sessions<br>▪ Motivation: Employees (N = 2,300) did a 1-day orientation introducing them to the new program and 200 supervisors received training in facilitating dialogue in the company<br>▪ Conversion: Intensive work on implementation. Each group had freedom on which activities to focus on (e.g., induction for newcomers, risk assessment)<br>▪ Maintenance: Introduced 2013 due to an increase in accidents. Employees who previously attended were invited to complete a refresher<br>▪ The program focussed on organisational/cultural changes based on interviews/observation/documentation<br>• Employees reported that managements intent/focus on health/safety and the new program led to successful implementation.
Roche et al., 2015 [24]	Alcohol use among workers in male-dominated industries: A systematic review of risk factors	• Risky alcohol use was associated with being male/middle aged, depression/negative life events, social norms at work (drinking norms), work conditions (high workloads/job stress, low collegial support), the team environment/dynamic, using alcohol to relax after work, and low SES.
Roche et al., 2016 [25]	Men, work, and mental health: A systematic review of depression in male-dominated industries and occupations	• Most studies reported higher levels of depression in male-dominated industries compared to the general population.
Ryan et al., 2017 [26]	A cross-sectional study of work-related and lifestyle factors associated with the health of Australian long distance commute and residential miners	• Recommendations from the report include better communication between workers/management (communication from the top-down and employees feeling they could raise concerns with seniors), training around mental health awareness, improved rostering (10 day-off roster, consecutive weekends), and continued professional support (in particularly peer mental health initiatives [mates in mining]).
Sinha & Vyas, 2018 [27]	Monitoring health and safety of oilfield workers through wearable technology	• Smart watches represent a cheap practical solution to monitor fatigue of workers.
Tynan et al., 2017 [28]	Alcohol consumption in the Australian coal mining industry	• 46% of males reported risky drinking behaviours but this was much lower for females (17%).<br>• Being younger, male, a smoker, using illicit substances, and higher levels of distress were associated with high alcohol use.
Yeoman et al., 2020 [29]	Health risk factors among miners, oil and gas extraction workers, other manual labor workers, and nonmanual labor workers, BRFSS 2013-2017, 32 States	• In oil and gas workers, prevalence of excessive alcohol use was 30% (similar to miners but higher than manual labourers).<br>• Prevalence of sleeping <7hrs was 39%.

**Health and wellness of fly-in fly-out workers**		
**Author, year**	**Title**	**Key findings**

Asare et al. 2021 [30]	Health and well-being of rotation workers in the mining, offshore oil and gas and construction industry: A systematic review	• Levels of psychological distress, depression, and anxiety varied across studies and levels of stress were categorised as low-moderate.<br>• Suicide risk was prevalent in around 25% of FIFO workers (Australia, onshore); however, the number of studies was low (N = 2).<br>• Job demands (e.g., workload, job stress), roster characteristics (roster type/length, shift length), work-home interference, increased remoteness, and workplace bullying/poor workplace culture were all associated with poor mental health, while social and managerial support were protective factors.<br>• Job demands and roster type were associated with more physical health complaints while social and managerial support was associated with less physical health complaints.<br>• Sleep duration and quality tended to be lower while workers were on-site. Sleepiness, insomnia, and fatigue were also prevalent in FIFO workers.<br>• Job demands and roster/shift patterns were associated with sleep and fatigue-related outcomes.<br>• In regards to lifestyle patterns, alcohol consumption and smoking was high and diet was poor, particularly while on shift, and physical activity levels were lower in offshore workers.
Asare et al., 2022a [31]	Multiple health-related behaviours among fly-in-fly-out workers in the mining industry in Australia: A cross-sectional survey during the COVID-19 pandemic	• During the pandemic, more FIFO workers smoked cigarettes and had risky alcohol consumption than the general population.<br>• FIFO workers completed more moderate-vigorous physical activity during the pandemic.
Asare et al. 2022b [32]	Health and related behaviours of fly-in fly-out workers in the mining industry in Australia: A cross-sectional study	• While on shift, sleep duration was reduced and fruit and vegetable intake was lower; however, smoking was similar to off-shift days, alcohol intake was lower, and physical activity levels were higher.<br>• Most participants had good physical health (91%), while one-third reported high-very high psychological distress (assessed via the K10).
Barclay et al., 2013 [33]	Factors linked to the well-being of fly-in fly-out (FIFO) workers	• Overall, physical and mental health was rated as good-very good in 75% of participants.<br>• Sleep disturbances (20%), feelings of loneliness/isolation (40%), and conflicting job and home/family demands (60%) were commonly reported in this cohort.
Bowers et al., 2018 [34]	Psychological distress in remote mining and construction workers in Australia	• In remote mining workers psychological distress was higher than the general population.<br>• 28% of participants reported high or very high levels of psychological distress compared to 11% of the general population.<br>• 22% of participants rated their mental health as poor or fair compared to 15% of the general population.<br>• Primary sources of stress included missing special events while on-site (87%), relationship problems with partners (68%), financial stress (62%), shift rosters (62%), and social isolation (60%).

		• Stigma of mental health problems (40%) and unavailability of help when needed (39%) were also causes of concern.<br>• Being stressed or extremely stressed about the stigma of mental health problems were significant predictors of high-very high levels of distress.
Crosscare, 2016 [35]	Challenges for FIFO workers in Australia	• Fatigue, poor workplace culture, strain on personal relationships, alcohol and drug use are often prevalent in FIFO workers which can impact physical and mental health.
Gardner et al., 2018 [36]	Mental health and well-being concerns of fly-in fly-out workers and their partners in Australia: A qualitative study	• FIFO workers reported (qualitative study) that their mental health/wellbeing were negatively impacted by being unhappy in FIFO work but not able to undertake lower paying employment, balancing work/life commitments, detachment from families, and a lack of social and employer support.
Gibson Smith et al., 2018 [9]	Health, self-care and the offshore workforce: Opportunities for behaviour change interventions, an epidemiological study	• Over 70% of offshore workers were classified as overweight or obese and 15% reported a long-term illness, of which 80% were taking medication.<br>• Median SF-8 scores were 56.1 (physical) and 54.7 (mental) representing high physical and mental quality of life.<br>• Over 50% were at risk of harmful alcohol use and 67% had poor sleep quality.<br>• 55% of participants met the recommended fruit and vegetable intake per day and 70% met the recommended physical activity guidelines.
Goater et al., 2013 [37]	Health promotion in FIFO and resident mine workforces: A case for a wellness-watch program	• Occupational hazards, lifestyle factors, and concerns such as substance abuse, sexually transmitted infections, depression, and other mental health concerns demonstrate the need for a holistic approach to improving the health of mine (in particular FIFO) workers.<br>• Mining should consider wellness monitoring/interventions which cover a range of factors.
Harris, 2016 [38]	The management of risk factors associated with FIFO workers' mental ill-health	• Poor mental health is the leading cause of absenteeism, poor work performance, and work turnover.<br>• Factors such as fatigue and being isolated (on-site) can worsen symptoms of psychological distress.
Joyce et al., 2013 [39]	Health behaviours and outcomes associated with fly-in fly-out and shift workers in Western Australia	• Shift work can lead to cardiovascular diseases and type 2 diabetes.<br>• FIFO workers are more likely to smoke and have higher levels of alcohol consumption.
Korneeva & Simonova, 2020 [40]	Job stress and working capacity among fly-in-fly-out workers in the oil and gas extraction industries in the Arctic	• Stress was higher at the end of the fly-in period.<br>• Health state and mood were higher at the beginning and middle of the fly-in period.
Labra et al., 2022 [41]	The overall health of men who do fly-in fly-out work in the mining sector	• Being away from their family was a significant cause of stress for FIFO workers and often fatigue, poor sleep, and changes in mood impact relationships once they return home.<br>• Physical health concerns of workers included physical inactivity while at work, and accumulated fatigue.

		• Despite reporting physical and mental health concerns, healthcare services were underutilised, particular supports relating to mental health (doctor, psychologist, social worker) with participants preferring non-professional supports.
Langdon et al., 2016 [42]	Australian fly-in, fly-out operations: Impacts on communities, safety, workers and their families	• FIFO workers are more likely to engage in unhealthy behaviours (excessive smoking/drinking) and be overweight or obese.<br>• Offshore workers are more likely to smoke, drink, have poor diets, and exercise less.<br>• Mental health remains stigmatised in the construction/mining industry with many unwilling to seek assistance.
Miller et al., 2020 [43]	Bullying in fly-in-fly-out employees in the Australian resources sector: A cross-sectional study	• 56% experienced workplace bullying and 32% reported moderate-severe depression.<br>• Having a supervisor who did not promote collaboration was associated with experiencing bullying.<br>• Bullying was associated with reporting clinical depression.
Parker et al., 2018 [44]	Impact of FIFO work arrangements on the mental health and wellbeing of FIFO workers	• Psychological distress was significantly higher in FIFO workers (33%) compared to the general population (10%).<br>• Sleep quality was significantly worse in FIFO workers and fatigue while transitioning from work to home was frequently reported.<br>• FIFO workers were more likely to report being bullied or witnessing bullying.<br>• Longer rosters were associated with poorer mental health and less time off between shifts increased stress and fatigue.<br>• Increased recreation and social opportunities (e.g., gym, pool tables), while on-site, were associated with better mental health.<br>• Families of FIFO workers also reported higher levels of psychological distress and poor mental health.<br>• Alcohol consumption and illicit drug use was higher in FIFO workers and they were more likely to take sleeping pills and smoke tobacco.<br>• Social support positively impacted mental health and wellbeing in both FIFO workers and their families.
Queensland Parliament Infrastructure, Planning and Natural Resources Committee, 2015 [45]	Inquiry into fly-in, fly-out and other long distance commuting work practices in regional Queensland	• Recommendations include minimum accommodation standards (relating to sleep/fatigue management, communication, access to recreation activities, healthy food, and social activities), and rostering which supports mental health, wellbeing and reduced fatigue.
Rebar et al., 2018 [46]	Health behaviours of Australian fly-in, fly-out workers and partners during on-shift and off-shift days: An ecological momentary assessment study	• Sleep quality was worse while on-shift.<br>• Workers and their partners had poorer nutrition and less time for exercise and relaxation on shift days.<br>• Workers and their partners tended to drink less alcohol on shift days.<br>• Workers and their partners smoked more during shift days.

Sustainable Built Environment National Research Centre, 2015 [47]	Challenges for the FIFO/DIDO workforce: Impacts on health, safety and relationships (Industry report)	• FIFO work led to depleted time/energy of workers, isolation, limited communication with family, workplace stress, concerns relating to shifts/rosters/work hours, and fatigue (particularly relating to travel between work and home).<br>• Recommendations for negating the impact of FIFO on health, safety and relationships included increased communication between workers and management, training (mental health awareness, family-work adjustments), better rostering (suggestion of a 10-day off roster), and external support (e.g., peer and organisational support/mentoring).
Torkington, Larkins & Sen Gupta, 2011 [48]	The psychosocial impacts of fly-in fly-out and drive-in drive-out mining on mining employees: A qualitative study	• There was limited awareness of EAPs and staff reported being unsure if nurses and medics on site were able to provide mental health support.<br>• In addition, those aware of professional supports available, including the EAP, still cited trusted friends or colleagues as their preferred source of support.
Turner & Rubin, 2022 [49]	Issues affecting mental health at a fly-in-fly-out mine site: A subjective impact ratings approach	• Lack of control at work, lack of sleep, missing home, and poor physical health were the strongest contributors to poor mental health.
Western Australia Legislative Assembly, 2015 [50]	The impact of FIFO work practices on mental health	• Rostering, fatigue, poor workplace culture, and strain on relationships/personal life are all significant stressors for FIFO workers.<br>• Recommendations included rostering which would support mental health/wellbeing, having controls in place to manage fatigue and related consequences, peer support programs be implemented.

**Healthcare delivery in the resource sector**		
**Author, year**	**Title**	**Key findings**
Adams, Lazarsfeld-Jensen & Francis, 2019 [51]	The implications of isolation for remote industrial health workers	• Nurses and paramedics working in onshore and offshore industrial health roles reported being unprepared for the broad scope of practice and isolation impacted their skill development/confidence to meet the requirements of the role.
Dugdale et al., 2022 [52]	Findings from a systematic review of fatigue interventions: What's (not) being tested in mining and other industrial environments	• Sleep in oil/gas workers improved with light therapy.
International Association of Oil and Gas Producers, 2019 [53]	Health management in the oil and gas industry	• Health management in the energy sector should encompass a management framework (leadership, risk management, continuous improvement, and implementation), health reporting and medical record management (legislative and internal reporting), and contractor health management systems.<br>• Health risk management should include assessments pertaining to fitness for work, health surveillance, worker welfare/wellbeing, waste management/food/water, occupational health, fatigue risk management, drug/alcohol misuse management, management of local environmental factors, and planning for pandemics/infectious disease concerns.

Ling et al., 2016 [54]	The economic impact of psychological distress in the Australian coal mining industry	• Lost productivity due to psychological distress (K10 scores) in the Australian coal mining industry was estimated at $154 million: hours lost x hourly wages.
Mosadenghrad & Langroudi, 2021 [55]	Health service delivery to oil companies' employees: A comparative review	• The type of service (medical emergency, occupational diseases, other), scope of the company's activity, and quality of the health service available in the region will dictate how healthcare and treatment are provided.
Norman & Valentine, 2020 [56]	Remote medicine: A textbook for trainee and established remote healthcare practitioners	• Remote health practitioners (offshore or remote) are generally trained as nurses or paramedics, supplemented by additional training to take on the expanded scope of practice.<br>• A remote healthcare system incorporates training in basic life support, effective medical communications, training for the remote practitioner and doctors providing support from a different location, and medical surveillance to pre-empt/detect avoidable medical events.<br>• Technology is crucial for medical care in the oil and gas industry: the technology allows decision support, collaboration, and sharing of medical records/data.
Tynan et al., 2016 [57]	Help-seeking for mental health problems by employees in the Australian mining industry	• Mine workers prefer non-professional mental health support.

**Healthcare in response to emergency and trauma situations**		
**Author, year**	**Title**	**Key findings**
Abhilash & Sivanandan, 2020 [58]	Early management of trauma: The golden hour	• Following an injury, the first 60 minutes (golden hour) is the most crucial window for dictating the outcomes for the patient.<br>• This period should encompass an assessment of the patient (e.g., whether a limb is compromised, major injuries) and resuscitation.
Acheampong & Kemp, 2022 [59]	Health, safety and environmental (HSE) regulation and outcomes in the offshore oil and gas industry: Performance review of trends in the United Kingdom Continental Shelf	• In the UK, health and safety in the oil and gas industry have improved between 1995 and 2018; however, low-probability-high-impact incidents are still a risk with considerable consequences.
Bonato et al., 2020 [60]	Medical emergency resource classification instrument in the oil industry, Brazil	• The risk instrument captures the risk scenario (administrative through to various industrial risks), accessibility (urban vs remote), and the population (number of workers) to provide an overall score.<br>• The score can then be used to determine which healthcare measures need to be in place (support coverage available [12h, 24h/nil per day], care staff [physician, nurse, or a combination], materials/drugs available]).
Croser, 2003 [61]	Trauma care systems in Australia	• Mining companies working in remote locations will train their own personnel to undertake rescue and first aid in industrial accidents in industrial complexes and mine sites.<br>• For trauma events in remote locations, retrieval by road or air is required.

Enright, Harman & Brune, 2016 [62]	Advanced life support in the mining environment	• All miners should be trained in CPR and AED.<br>• Mines further from emergency medical care or the local hospital should have more staff on-site able to provide medical care/first aid.
Fatovich et al., 2011 [63]	A comparison of metropolitan vs rural major trauma in Western Australia	• Risk of major trauma death is more than double in rural/remote WA than metropolitan.<br>• If patients survive until emergency retrieval, mortality rates are similar to those in the metropolitan area.
Gibson-Smith et al., 2019 [64]	Medical evacuations and work absences in offshore oil and gas industry personnel	• 20% of respondents were unable to travel offshore for work due to health-related problems (over the course of their careers).<br>• Absence was due to injury (39%), short-term illness (25%) and long term illness (5%).<br>• 12% had required medevac and this was primarily from injury (34%) or short/long-term illness (32%).
Huzaini et al., 2020 [65]	Exploring of offshore medical emergency response system challenges in oil and gas environment	• Challenges to emergency response related to communication (quality of instructions as well as phone/email/radio which facilitates communication), decision making (stakeholders on site as well as medical support located elsewhere), layout of the facility (e.g., moving patients from the accident site to the helipad), logistical problems (emergency transport is outsourced, meaning that staff on-site have limited control of timelines when waiting for patient transport/extraction), offshore medic responses (inexperience, workload, poor reporting of the offshore medics), protocols (additional procedures/company protocols may delay treatment), and weather conditions (effects time taken to respond in an emergency situation).
Ims, 2013 [66]	Emergency preparedness in Arctic oil and gas exploration	• Limited helicopter resources present a major concern.
International Association of Oil and Gas Producers, 2022 [67]	Medical emergency response and primary healthcare guideline	• Healthcare strategies should be based on worksite risks, healthcare needs, and locally available resources.<br>• Healthcare provision should be able to be scaled up or down as needs change.<br>• Where needs for healthcare are lower, additional activities such as health promotion (physical/mental health or wellbeing), primary healthcare, or occupational health services, can be conducted.<br>• Medical emergency response:<br>▪ Designed to minimise harm and optimise recovery<br>▪ Appropriate care relates to quality of care and speed of healthcare delivery, which is particularly relevant for more remote areas.<br>▪ MER is separated into four tiers:<br>▪ First aid (tier 1): including AED by a first responder, to be delivered in 3-5 minutes (as a guide).<br>▪ Assessment, stabilisation, treatment (tier 2): delivered by a trained emergency professional (doctor/first responder) as soon as practical, depending on risks on site, recommended delivery 20-60 minute

		▪ Admission/treatment (tier 3): Admission to the most appropriate hospital as soon as possible, recommended within 3-4 hours of the emergency. If access to care as part of tier 3 is likely to be delayed (e.g., increased remoteness) then tier 2 should be strengthened (better diagnostic/treatment capabilities or more specialised staff).<br>▪ Referral to a specialist hospital (tier 4): As soon as possible and only if appropriate. Tier 4 may not always be required.<br>▪ Additional strategies need to be in place for staff who are alone to alert the need for medical attention, if required: includes CCTV, radio check-ins, wearing monitoring devices, or panic buttons.<br>▪ Worksites where a tier 3 (hospital) facility cannot always be reached within 3-4 hours requires tier 2 to be strengthened. This includes overall remoteness as well as remoteness from specific treatments (e.g., the closest hospital does not have a cardiac unit).
Ponsonby, Mika & Irons, 2009 [68]	Offshore industry: Medical emergency response in the offshore oil and gas industry	• Medical emergency response is generally broken into tiers (1: basic first aid, 2: Advanced first aid, 3: trained medic paramedic/emergency medical technician.4: doctor/nurse in a primary care facility, 5: specialist doctor in a secondary/tertiary facility).<br>• Management of emergency response in a remote site generally involves an on-site practitioner providing care based on observation on-site with access to a remote supervisor who can provide advice and grant authority for specific procedures/drug administration.<br>• Onshore staff may be working alone for a period of time before support can be provided (directly or remote) which necessitates further training for these staff.<br>• On-site facilities should house equipment and drugs which can meet limited primary care needs/minor ailments, as well as enough medical items to provide emergency care stabilisations for serious illness/injury. Stock levels and medication (e.g., expiries) should be closely monitored.<br>• Telemedicine provides a platform to move information rather than the patient (i.e., deliver health care before or instead of emergency retrieval).<br>• Telemedicine may be used to seek a first opinion (e.g., X-ray or ECG interpretation) or second opinion (define whether a case is an emergency, define level of urgency, or seeking advice on treatment before an evacuation).<br>• Telemedicine requirements include medical equipment (equipment with capacity to share data/information and communicate visually via video link), communication systems (e.g., satellite), platforms/systems which facilitate data exchange/interpretation/record keeping, and sufficiently trained medical personnel (on and off-site).
Sae-Jia & Sithisarankul, 2020 [69]	Medical evacuations among offshore oil and gas industries in the Gulf of Thailand	• Between 2016 and 2019 there were 416 evacuations.<br>• 84% of evacuations were due to illness and 60% were considered unpreventable/difficult to prevent.

Toner et al., 2017 [70]	Medical evacuations in the oil and gas industry: A retrospective review with implications for future evacuation and preventative strategies	• Between 2008 and 2012, 130 medevacs were conducted (4 per 1000 population).<br>• Rates were higher for high/extreme risk countries.<br>• Reasons for medevac were mostly trauma, digestive issues, musculoskeletal, cardiac and neurological.

**Primary healthcare**		
**Author, year**	**Title**	**Key findings**
International Association of Oil and Gas Producers, 2022 [67]	Medical emergency response and primary healthcare guideline	• Healthcare strategies should be based on worksite risks, healthcare needs, and locally available resources.<br>• Healthcare provision should be able to be scaled up or down as appropriate.<br>• Where needs for healthcare are lower, additional activities can be conducted such as health promotion (physical/mental health or wellbeing), primary healthcare, or occupational health services.<br>• Primary healthcare:<br>▪ Can be driven by optimal utilisation of tier 2 resources, legislative requirements, health risks assessments, or insufficient local primary healthcare.<br>▪ Models of primary healthcare generally fall under capacity building of existing healthcare (added equipment/facilities) or establishing an on-site/near site practice to deliver primary healthcare.<br>▪ Capacity building involves training/retraining local clinicians, purchasing equipment, etc to increase diagnostic capabilities (including telehealth).<br>▪ Establishing primary health care using a 'sole proprietor' model involves a clinic which is built/operates independently. Under this model the clinic needs to be able to deliver primary healthcare as well as have a system in place to refer more complex cases.<br>▪ Primary healthcare may also be established by a company using a conglomerate model (partnership with another private company). Benefits include cost-effectiveness, ensuring adequate workload and skills of staff, and benefits to the local community in addition to the company.<br>▪ Primary health care should include wellbeing alongside traditional primary healthcare.

**Preventative healthcare: Organisation-led interventions**		
**Author, year**	**Title**	**Key findings**
Asare-Doku et al., 2020 [71]	Mental health interventions in the mining industry: A narrative review	• Contributors to poor mental health in the mining industry include being male dominated, working in remote locations, rostering, and commute type (e.g., FIFO/DIDO).

		• Mining has a higher prevalence of mental health concerns than other industries, as well as reports of alcohol and drug abuse.<br>• Mine workers are often less likely to seek help for mental health, relating to stigma and masculinity within the male-dominated industry.<br>• Among miners in Poland, a mindfulness-based intervention was able to reduce anxiety and depressive symptoms. A mindfulness app, used by miners in Australia for 30 days, also led to improvements in depressive/anxiety symptoms.<br>• In a study of miners in North Queensland, employees lacked awareness of employee assistance programs (EAP). Those with knowledge of the EAP still reported preferring to seek support from other sources (colleagues/family).<br>• Peer support interventions showed consistent positive results:<br>▪ A peer-based mental health program led to an increase in workers' ability to identify employees with mental health problems.<br>▪ Help seeking behaviours and attitudes to mental health improved after a mental health program (A peer support suicide prevention program based on Australian evidence).<br>▪ Employees in multiple studies reported that workplace commitment to good mental health or perceived positive organisational support improved employee mental health, well-being, and work output.
Bezzina et al., 2021 [72]	Health and wellness in the Australian coal mining industry: A cross sectional analysis of baseline findings from the RESHAPE workplace wellness program	• Participants: 90% male, 59% aged 25-44 yr, 19% healthy weight/41% overweight/39% obese.<br>• Mine workers are more likely to be overweight/obese compared to the general population.<br>• Being in a rural/regional area, as well as shift work, may be risk factors, both of which are common in mine workers.
Campbell & Burns, 2015 [73]	Total worker health: Implications for the occupational health nurse	• Total Worker Health (TWH; NIOSH) aligns occupational health and safety with health promotion strategies to prevent worker injury/illness and improve health/well-being.<br>• Models which combine occupational health services with primary care are also encouraged.<br>• Workplace wellness programs improve worker health and control healthcare expenses.<br>• Essential elements of TWH include commitment from management, safety culture, health culture, hazard recognition training, and worker empowerment.<br>• CDC reported that TWH can lead to a decrease in health care costs and increased employee productivity.<br>• Core drivers of excess health care costs and lost productivity relate to obesity, physical inactivity, and tobacco use.
Fagade et al., 2008 [74]	Shifting focus in workplace health care services delivery strategy: The experience of Chevron Nigeria	• Historically, healthcare for oil/gas workers has focussed on disease management rather than preventative health care.<br>• Periodic health screening services revealed high rates of chronic disease including hypertension (20%), overweight/obesity (50%), lipid disorders (18%), and diabetes (5%).

		• Based on the rates of chronic disease, it was proposed that chronic disease management was likely of interest to the company, particularly via lifestyle/behaviour modification, and that addressing these concerns could lead to a considerable reduction in healthcare costs.<br>• To effectively transition from disease management to preventative healthcare, organisational changes were made, with programs implemented relating to physical activity interventions, web-based health trackers for employees (personal use), health monitoring station (BMI, Blood Pressure), and health education/literacy.<br>• Challenges related to addressing the broader range of health determinants, supporting employees to be proactive with their health, and creating buy-in with the various initiatives.
Khanal et al., 2016 [75]	Evaluation of the implementation of Get Healthy at Work, a workplace health promotion program in New South Wales, Australia	• Get Healthy at Work is a state (NSW) program available to all businesses. It uses a five-step health promotion strategy available from a service provider (the organisation) or self-directed DIY portal.<br>• The program includes a needs assessment, action plan, implementation, and progress monitoring/review.<br>• The program is being simplified/revised.
Feltner et al., 2016 [76]	The effectiveness of Total worker health interventions: A systematic review for a National Institutes of Health pathways to prevention workshop	• Health behaviours such as diet, physical activity, and tobacco use can be improved via Total Worker Health-related interventions.
National Institute for Occupational Safety and Health, 2016 [77]	Fundamentals of total worker health approaches: Essential elements for advancing worker safety, health, and well-being	• Total Worker Health is a collection of policies, programs, and practices integrating protection from hazards and prevention of injury and illness to advance worker wellbeing.<br>• May cover various aspects including physical, biological and psychosocial hazards, organisation of work, compensation/benefits, and work-life integration.
Sayers et al., 2019 [78]	Does help seeking behavior change over time following a workplace mental health intervention in the coal mining industry?	• Following a peer support mental health intervention, employees were more confident and more likely to seek professional and non-professional support for mental health.<br>• Participants preferred face-to-face delivery of mental health training.<br>• Prior to the intervention, more employees felt that friends/colleagues would treat them differently if they found they had mental illness, and that they would be treated poorly in the workplace.<br>• Friends/family were the most preferred sources of support even after the intervention.
Tynan et al., 2018 [79]	Feasibility and acceptability of strategies to address mental health and ill-health in the Australian coal mining industry	• The higher prevalence of mental health concerns in miners may be related to long work hours, rostering, shift work, physically demanding and repetitive tasks, working long distances from home, and displacement from family and other social support networks.<br>• The intervention involved training and establishment of a peer-based network.<br>• Following the intervention, participants exhibited greater confidence in identifying workmates with mental health problems, knowing where to find support, willingness to start a conversation, and perceptions of workplace mental health support.

Telehealth and telemedicine		
**Author, year**	**Title**	**Key findings**
Bouabene, 2002 [80]	Providing emergency medical care to offshore oil and gas platforms in the Gulf of Mexico using telemedicine	• Utilisation of high-resolution video conferencing allows patient visits to be conducted in real-time, assists with decision making about the need for more extensive treatment, and can reduce unnecessary evacuations.<br>• 39% of patients from offshore facilities treated in the emergency department could have been treated through telemedicine without being evacuated.
Anscombe, 2010 [81]	Healthcare delivery for oil rig workers: Telemedicine plays a vital role	• Of 54% of patients who received telemedical services, >98% were satisfied with the care they received.<br>• Telemedicine improved morale/welfare, increased perceived productivity of workers, and made workers feel that the company cared about them working in the remote environment.
Berg et al., 2015 [82]	Remote health care: A game changer for the Arctic	• The remote healthcare strategy identified risks (distance from care, high risk evacuations, deviations consistently required), key strategy components (planning/prevention, people/competency/mindset change, equipment/supplies, communication/telemedicine, and collaborations), and benefits (better diagnostic/health outcomes, minimise unnecessary/high risk evacuations, reduced operational downtime, evidence of innovation).<br>• The healthcare solutions involved a virtual doctor's office linked to a hospital, which was enhanced by medical technology on-site (lab testing equipment, X-Ray, and ultrasound equipment. This allowed the physician on board to communicate with onshore specialists. The strategy extended to fitness to work, drug/alcohol, hygiene practice, and food/water safety.
Boniface et al., 2011 [83]	Tele-ultrasound and paramedics: Real-time remote physician guidance of the focused assessment with sonography for trauma examination	• Paramedics without previous ultrasound experience were able to capture images in a trauma situation with remote guidance in less than 5 minutes.
Cheskes et al., 2020 [84]	Improving access to automated external defibrillators in rural and remote settings: A drone delivery feasibility study	• AED delivery by drone decreased response time in out of hospital cardiac arrest.
Dehours et al., 2021 [85]	User satisfaction with maritime telemedicine	• Evaluated a 24-hr emergency physician service, affiliated with a teaching hospital and delivered via phone/email: on-board caregivers support the service.<br>• A medical observation sheet is prefilled prior to the consultation. The patient is generally supported/treated/watched onboard via repeated teleconsultations but are evacuated if necessary.<br>• 84% of respondents felt the wait time was satisfactory/very satisfactory.<br>• 97% were satisfied/very satisfied with the relationship with their physician.
Dittrick, 2009 [86]	Offshore care on line	• Remote healthcare can be improved via telehealth and onsite equipment.

		• Patients were satisfied with this model of care and believed that use of video improved the quality of the visit.
Eadie et al., 2018 [87]	Remotely supported prehospital ultrasound: A feasibility study of real-time image transmission and expert guidance to aid diagnosis in remote and rural communities	• Paramedics were supported via satellite communications to conduct an ultrasound in a simulated trauma situation.<br>• In 94% of simulated trauma scans, appropriate images were obtained.
Evans et al., 2016 [88]	An innovative approach to enhancing access to medical screening for miners using a mobile clinic with telemedicine capability	• In the US, incidences of various respiratory diseases in miners are increasing, which necessitates early screening and intervention in this at-risk group.<br>• Miners represent an underserved and medically vulnerable group.<br>• Mobile screening with telemedicine means that miners can be seen in an hour long visit on-site and any required follow-up can be organised via a telehealth model to ensure ongoing communication/follow-up/access to care.<br>• All miners who participated in the mobile screening clinicals rated it as very good (92%) or good (8%).<br>• Miners participating in in the clinic felt that they were receiving care not available in their own communities due to a lack of specialist services.
Evjemo, Reegard & Fernandes, 2015 [89]	Telemedicine in oil and gas: Current status and potential improvements	• Companies reported changes in healthcare delivery for offshore workers related to improving the quality of the services, which was achieved via including medical experts in clinical decision making.<br>• To ensure healthcare-related decision making was more efficient, investments were made in better tools for communication/collaboration such as video conferencing and more medical equipment offshore, which enabled data to be shared with experts onshore.<br>• Challenges of changing the healthcare models related to managing the complexity of the different systems which needed to be integrated (offshore/onshore medical professionals, patient transport, equipment for collaboration and medical examinations, rules/regulations, and past work behaviours/preferred ways of working).
Hellfritz, Waschkau & Steinhauser, 2021 [90]	Quality indicators of telemedical care offshore – A scoping review	• No validated indicators of telemedical care offshore were identified in the review but possible indicators were identified which should be further explored:<br>▪ Reliable, transmittable continuous equipment-based monitoring offshore available within 15 min<br>▪ 12-lead ECG<br>▪ High-quality video systems available with 15 min<br>▪ Telemedically accessible electronic health records<br>▪ major hospital available for specialist consulting<br>▪ appropriately qualified teleconsultant physicians<br>▪ Qualified offshore medical personnel<br>▪ Defined protocols for most common medical cases

		▪ Communication of changes to telemedical care<br>▪ Training of all personnel in using telemedicine.
Islam et al., 2019 [91]	Portable health clinic: An advanced tele-healthcare system for unreached communities	• The portable health clinic system includes a box containing variety of medical sensors/tablet/printer, a health worker, an online server for data storage/sharing, and a remote call centre (doctors on staff).
Latifi, 2020 [92]	Telemedicine for trauma and emergency care management	• Telemedicine can assist with limiting the disparity in trauma care between rural/urban centres and could cover care across the entire spectrum from injury site (pre-hospital care), site where the patient is seen (rural hospital), and the trauma centre/tertiary hospital.<br>• When trauma surgeons/trauma care providers offer advice over the phone, they often play it safe by transferring the patient, which may delay treatment (until they reach the referral centre). However, with teletrauma services, the consultant can see the patient and obtain objective data (e.g., vitals) to make better decisions and recommend care that could be delivered before or instead of transfer.
Mika et al., 2009 [93]	E-health in an international oil and gas company – Saipem's experience	• E-health implemented as part of day-to-day operations encompasses health care, health database, education/training, and work care<br>▪ Health care: 24h 'call centre' which connects employees to a doctor via telephone, 24h telemedicine service for cardiological emergencies, collaboration between health services via telehealth.<br>▪ Health database: Comprehensive medical/health data (which is confidential) along with software used to show real time medical stocks (consumables/medication).<br>▪ Health education: Company-wide intranet health portal with resources for employees (health literacy, workplace requirements) and medical personnel (health standards/guidelines) and an e-learning platform with educational material (health, OH&S).<br>▪ Work care: Site doctor is responsible for the health of the employee and decides future management of a patient.
Stilz et al., 2022 [94]	A prospective investigation of the impact of telemedicine and telemetry on global medical evacuation rates	• Real-time video (81%), vital signs monitor (31%), text messages (14%), and uploading images/electronic medical records (12%) were the most used communication methods/digital equipment.<br>• The most common digital remote tools used were camera (57%), video (32%), digital stethoscope (29%), and video-assisted otoscope (16%).<br>• For locations without telehealth capabilities, the odds of medical evacuation were significantly higher compared to where telehealth was available (OR = 2.9-3.6).
Visionflex, 2022 [95]	How clinical telehealth helps isolated FIFO teams	• For remote workers, telehealth allows clinical care to be delivered on site.<br>• The telehealth system captures images, videos, and patient vitals during the consultation which, when combined with collaboration with experts, improves decision making (accuracy and timeliness) and appropriate treatment can be initiated earlier.

		• Telehealth improves work outcomes and patient satisfaction while reassuring workers of their safety by distilling knowledge of additional safety measures while they are at work.
Webster et al., 2008 [96]	A low-cost decision support network for electrocardiograph transmission from oil rigs in the North Sea	• 14 oil rigs were supplied with thrombolytic drugs and paramedics on the rig were trained to deliver them.<br>• Companies also invested in ECG machines which allowed electronic transmission via email.<br>• Over a 3 year period, 47 cases of chest pain were handled via telemedicine and only 13% of these were airlifted to shore.
Woldergay, Walderhaug & Hartvigsen, 2016 [97]	Literatures review of telemedicine services in maritime and extreme weather	• Success of telemedicine in offshore contexts can be poor due to poor communication networks, bad weather, and longer time/distance from helicopters and medical evacuations.
Woldaregay, Walderhaug & Hartvigsen, 2017 [98]	Telemedicine services for the Arctic: A systematic review	• In terms of telemedicine, satellite is the most used form of communication followed by mobile.<br>• Telemedicine modalities largely include video (27%), still pictures (25%), audio (19%), and text (e.g., email; 17%).<br>• Telehealth is most commonly used for data sharing/decision making (27%), consultation (24%), and radiology (22%).<br>• Telehealth should be used to support, rather than be a solution for, remote accident/emergency response.

**Public-Private Partnerships in Healthcare**		
**Author, year**	**Title**	**Key findings**
ASMOF Doctors' Union, 2021 [99]	The Doctors' Union calls for an end to public private partnerships	• Called for the NSW government to remove PPPs in hospital planning.<br>• Suggest that the private operator invested insufficient resources in setting up the hospital.
Basabih, Prasojo & Rahayu, 2022 [100]	Hospital services under public-private partnerships, outcomes and, challenges: A literature review	• PPPs combine advantages of the private (innovation, knowledge, skills, efficiency, and entrepreneurship) and public sectors (responsibility, social justice, public accountability, quality infrastructure, and quality services).<br>• PPPs can be divided into four models:<br>▪ Infrastructure: design, build, finance, operate, lease, and transfer models.<br>▪ Service delivery: contracting in and out management contracts, co-location and franchising.<br>▪ Financial protection model: vouchers, health cards, insurance, and conditional cash incentives.<br>▪ Other: public-private mix, telemedicine, social marketing/health, training, research, and capacity building.<br>• PPPs have consistently shown improved hospital performance (patients seen, diagnosis and referral rates/times, length of stay, patient satisfaction, etc.).

		• Cost effectiveness of PPPs in a hospital context have shown variable results.<br>• Challenges include government regulations, inadequate resources, poor communication between public/private sectors, negotiating shared risk between public/private partners, and inadequate monitoring/evaluation.
Bendigo Health, 2023 [101]	Public Private Partnership	• Bendigo hospital: public sector is responsible for operating the hospital/core clinical services and teaching/training/research.<br>• Private sector is responsible for design, construction, building maintenance, non-clinical services (meals, cleaning, porter, car park management, grounds maintenance, security).
Eurodad, 2022 [102]	History repeated: Why public private partnerships are not the solution	• PPPs are promoted as overcoming challenges in financing, implementation and delivery of infrastructure and public services. PPPs assume that the private sector brings additional funding and efficiency.<br>• Concerns surrounding PPPs include costs associated with contract negotiations and renegotiations which counteract any additional profitability.<br>• Issues arise where private organisations are accountable to stakeholders not citizens/communities.
Farquahar, Moran & Schmidt, 2020 [103]	Mechanisms to achieve a successful rural physiotherapy public-private partnership: A qualitative study	• Physiotherapy was provided to hospital inpatients/outpatients and aged care residents and the health service provided treatments rooms for physiotherapists to see patients.<br>• Success of the model was defined as improving access to healthcare for patients and satisfying stakeholders (partner organisations, communities, GPs, and patients). All participants reported it as being successful.<br>• Mechanisms for achieving success included provision of human/other resources for the workforce model and partnership, stakeholder engagement, and streamlined processes for content of the contract, administration, managing private employees in a public setting, and communication.<br>• The workforce model was seen as an enabler by public and private participants: nursing staff could carry out recommendations so that therapy was provided more frequently (between visits) and an allied health assistant also contributed to this but was more cost effective, the model relied on appropriately skilled therapists to cover a broad scope of practice.<br>• Success of the partnership relied heavily on motivation and consistency of stakeholders whereby the arrangement needed to be equally beneficial for public/private, and turnover of staff created challenges.<br>• The nature of the contract was also important: it was perceived that the contract would enable appropriate service provision, outline responsibilities, and ensure appropriate services. Referrals could only be made by the managers to avoid therapists making their own referrals/choosing patients, an hourly-rate-for-service model was used whereby time was invoiced for time allocated to the patient to enable flexibility and adjustments for service demands: this created flexibility in work arrangements for the physio (public

		perspective) but the ad hoc nature made it difficult schedule time to see inpatients on the same day as the referral.<br>• Administrative burden in contract management/accounting was a concern and it was a lengthy negotiation to finalise the contract.<br>• A significant barrier was managing private physiotherapists in a public setting due to uncertainty in responsibility for managing the service, ensuring compliance/fit-for-purpose, and managing contractors.
Ganapathy & Reddy, 2021 [104]	Technology enabled remote healthcare in public private partnership mode: A story from India	• To ensure PPP sustainability, it should be developed using building of trust, clearly defined objectives/roles, time commitment, transparency/candid information (particularly risk/benefit), contract flexibility, technical assistance/financial incentive for procedural arrangements, and awareness of structural changes related to responsibility and decisions.
Hall, 2015 [105]	Why public-private partnerships don't work: The many advantages of the public alternative	• Raises the viewpoint that private companies seeking to make profits is incompatible with ensuring universal access to public services.<br>• Value for money: Cost of capital is cheaper without a PPP, transaction costs of tendering add 10-20% to costs, PPPs are risky to the public sector (incomplete contracts, likelihood of renegotiations, liabilities in the case of bankruptcy of the private organisation).
Infrastructure Partnerships Australia, 2013 [106]	Response to: Future direction for Victorian PPPs	• The PPP model has been mostly positive for the public sector with independent research showing reduced construction costs/time (average of 11.5% cost saving).<br>• PPPs may lead to increased efficiency/quality and possible innovative models of service delivery.<br>• Full-service PPPs have had mixed results<br>• A competitive process for PPPs can lead to cost efficiencies of up to 25% and improved service quality and flexibility of services.<br>• PPPs are generally successful when they create alignment between public/private outcomes, financial incentives for on-time delivery, design innovation, and operational efficiency.<br>• Accepting PPP bids based primarily on cost may lead to poorer outcomes: factors such as the potential for innovative quality service delivery should be prioritised.<br>• Service PPPs relate to models which engage not-for profit, non-government, and private providers in the delivery of public services.<br>• Performance based contracting drives service improvements<br>Case study: La Trobe Regional Hospital:<br>• After six months of operation, the private operator requested additional funding from the Government.<br>• Two years later, operations were returned to the public sector with the private operator required to pay approximately $2 million.<br>• The private partner was losing money due to an underestimation of staffing, incorrect tax assumptions (e.g., assuming that tax exemptions relevant to public hospitals would

		apply), failure to understand funding of public hospitals, underestimation of costs, and assuming that the Government would be willing to renegotiate the contract.<br>• Highlights the need to determine value for money, risk transfer, and accounting treatments pre-bid.<br><u>Case study: Port Macquarie Base hospital</u><br>• First public hospital delivered via a PPP: the Government became a health service purchaser rather than provider.<br>• The private organisation was responsible for design, construction, financing, and operation.<br>• The PMBH underperformed on all key indicators and the Government bought back the hospital seven years ahead of the planned end date.<br>• The contract for this PPP did not adequately address incentives, risk/rewards, and the operator was inexperienced.
Institute for Global Health Sciences, 2018 [107]	PPPs in healthcare: Models, lessons, and trends for the future	• Characteristics of PPPs include a long-term contract (typically 15+ but may be 5+, allocation/shared risk, performance indicators (mutually agreed upon), and government ownership of assets (facilities/equipment at the end of the contract).<br>• Historically, governments have engaged the private sector through PPPs to finance/co-finance a project, design infrastructure/care delivery models, build facilities, maintain infrastructure, operate nonclinical services, and/or deliver clinical services and support services.<br>• Types of healthcare PPPs:<br>▪ Infrastructure-based model: To build/refurbish public healthcare infrastructure.<br>▪ Discrete clinical services model: Add/expand service delivery capacity.<br>▪ Integrated PPP model: Provide a comprehensive package of infrastructure and service delivery.<br>• Typical PPP stages:<br>▪ Identify a need: Public sector identifies need based on local health/national strategy, this need requires private sector resources or skills.<br>▪ Delivery options considered: Feasibility/economic studies are conducted to identify potential options, options ranked to select the preferred delivery option, and legislation/environment and policies are reviewed.<br>▪ Competitive tender issued: Public sector issues tender documents.<br>▪ Contract awarded: Contract terms negotiated, private partner secures funding to deliver the project.<br>▪ Initiate operations: Build activities initiated as per contract, implementation and operations begins, contract management required for the life of the project.<br>• In integrated models, the private partner is responsibility for facilities and non-clinical services (e.g., housekeeping, utilities) after construction.

		• For discrete/integrated, the private partner may offer primary care through to specialty services. The private partner is also responsible for human resources/staffing.<br>• In healthcare PPPs, all facilities and services revert to the public sector at the end of the project to ensure the service is maintained and delivered at the required standards.<br>• Risks should be shared between private/public partners and typically relate to general/financial risk, planning/design/construction risk, and operating risk/clinical performance.<br>• While less common, the private partner may present a proposal to the public sector.<br>• PPP contracts create a relationship between payments and performance, with payments used to incentivise the private partner to meet desired outcomes:<br>▪ PPPs generally use a single payment mechanism to cover both infrastructure/services.<br>▪ The public sector generally will not make any payments unless the contract terms are met.<br>▪ Projects including clinical service delivery involve more complex arrangements.<br>• Required for PPP success:<br>▪ Political will.<br>▪ Transparency: Strong partnership with shared goals.<br>▪ Broad stakeholder engagement.<br>▪ Contract completeness, flexibility, and governance: Clear terms which support corrections and meet changing needs of the population served.<br>▪ Legislative and regulatory framework.<br>▪ Public sector capacity.<br>▪ Private sector capacity.<br>▪ Fiscal space.<br>• Key recommendations for best practice PPP management in healthcare:<br>▪ Ensure PPP policy and legislation is robust and consistent with other policies.<br>▪ Prepare an evidence-based delivery plan.<br>▪ Establish procurement protocols and documents.<br>▪ Develop a specialist unit to manage the program.<br>▪ Plan program management resources/training.<br>▪ Ensure political and civil service support.
Jefferies, Gajendran & Brewer, 2013 [108]	Public private partnerships: The provision of healthcare infrastructure in Australia	• Uses the Newcastle Mater Hospital redevelopment project as a case study to discuss the establishment and rationale for the PPP.<br>• At the time of publication previous PPPs (hospitals) in Australia had demonstrated limited success (Port Macquarie base hospital, Latrobe regional hospital, Berwick community hospital).

		• Focussed on redevelopment of the hospital which due to its age was not appropriate for delivery of many services (e.g., cancer care, emergency). Substantial upgrades were required which exceeded government funding.<br>• Risk areas identified:<br>▪ Quality of service/hospital.<br>▪ Timely delivery/costs within budget.<br>▪ Disruptions to hospital activities.<br>▪ Urban development.<br>▪ Equality/availability of opportunity, and information and consultation.<br>• 28 year project involving financing/design/construction/commissioning for new buildings, refurbishment of old building, transfer of local mental health services, maintenance of buildings/car parks, utilities, and management services as well as non-clinical services.<br>• Proposed revenue was on a monthly basis dependent on performance beginning when the hospital was operational.
Joudyian et al., 2021 [109]	Public-private partnerships in primary health care: A scoping review	• Contracted primary health services included maternal/child health care, family planning, environmental health, school health, health education, immunisation services, health promotion, postpartum services, among others were outsourced to specific target groups (including industrial workers).<br>• Primary care was typically contracted to the private sector to facilitate healthcare access/coverage for the community: Most outcomes were positive but not all the time.<br>• Challenges to PPPs for primary healthcare:<br>▪ Education<br>▪ Management<br>▪ Human resources<br>▪ Financial resources<br>▪ IT systems
Makinen, 2000 [110]	Working with local communities and health care systems in areas of oil and gas operations	• Framework presented is based on real-world examples (international data).<br>• Oil and gas companies seeking to work with local health services should consider stakeholder consultation/ownership (acting with consent and support of local stakeholders), sustainability (changes made in the local health system should be sustainable within local resources), and the leveraging of resources (oil/gas companies stimulating the contributions of others who are directly responsible or interested in the health systems overall).<br>• Phases involved in working with the health services should include sizing up the situation (gathering information concerning the health situation as it affects the oil and gas companies), situational analysis (evaluate system strengths/weaknesses and opportunities for intervention that would be feasible in light of resourcing), and formulating a strategy

		(oil/gas companies and local healthcare authorities/providers should be jointly involved in this process).
Tabrizi, Azami-Aghdash, Gharaee, 2020 [111]	Public-Private Partnership policy in primary health care: A scoping review	• The private sector sees PPPs as an opportunity for growth/profit while providing facilities and innovation for the public sector.<br>• The private sector benefits from efficient and cost-effective mechanisms for implementing their goals and policies.<br>• Under successfully implemented PPPs, the service users are expected to receive a greater diversity of coverage, higher quality services, better access, and lower costs, resulting in better health and satisfaction with the service.<br>• In PPP models for primary health care, the private sector is primarily responsible for service provision.<br>• Most studies show that PPPs have a positive impact on primary health care.
Weinberg et al., 2002 [112]	Using the oil and gas producers guidelines on strategic health management: Achieving sustainable healthcare improvement	• Principles and guidelines for incorporating community health into project planning/management emphasises cooperation among oil and gas companies, local government, and social agencies.<br>• Industry cooperation benefits industry and the host community, industry can help local governments to meet their responsibility for community health.<br>• A focus on primary health care will have the greatest impact.<br>• Stakeholder engagement/consultation should occur early.

**Resource sector impacts on the local community and access to healthcare**		
**Author, year**	**Title**	**Key findings**
House of Representatives Standing Committee on Regional Australia, 2013 [113]	Cancer of the bush or salvation of our cities	• Concerns were raised regarding how FIFO workers impact the fly-in community they come to (funding, resources, healthcare, etc), that the workforce should integrate with the local community, and that in DIDO regions, road safety is a serious concern (for workers and the community).<br>• FIFO workers impact the provision of local medical services: around a third of patients seen in mining towns are often from a location outside of the catchment area and many doctors reported reduced capacity to meet the health needs of residents, as a result.
Becker, 2018 [114]	Help wanted: Health care workers and mental health services: An analysis of six years of community concerns from North Dakota's oil boom residents	• Community members in towns experiencing an influx of oil workers frequently express concerns regarding reduced access to healthcare and mental health services.
Constantine & Battye, 2015 [115]	Mining towns – does the boom mean bust for health services	• Growth in the mining sector benefits local communities via job creation and economic benefits.<br>• This growth, however, can create increased demands on health services.

		• 25-30% of health service presentations in the area (Moranbah) were from non-residents, with the proportions of non-resident patients increasing over a five-year period.<br>• In 2011 almost one-third of ED presentations were by non-resident patients, of which 50% lived in work camps.<br>• Almost half of the presentations by non-residents were non-urgent.<br>• Demand on mental health services also increased with 25% of these patients being non-residents.<br>• There are limited medical/allied health practitioners in the region with specific skills in occupational medicine, which impacts return to work strategies.<br>• There was a significant lack of access to radiology services.<br>• Non-residents generally had a poorer understanding of the health services available than residents, including the breadth and limitations of health service provisions in the area.<br>• Much of the hospital presentations by non-residents could have been managed by a GP.
Rifkin et al., 2015 [116]	Benefits and burdens for rural towns from Queensland's onshore gas development	• In communities where workers come for mining and/or oil and gas work, the community may experience deficits in health care due to increased demand/utilisation.<br>• It is suggested that the heavy reliance of FIFO work in mining and oil and gas may mean that economic benefit seen by mining booms does not remain in that area and benefits are often inconsistent (e.g., boom/bust cycles).
Sigal, 2016 [117]	Socioeconomic effects of oil drilling: The case of Ecuador	• For locals, oil drilling reduces the quality of employment and healthcare; however, infrastructure and schooling are improved.
Witt & Simpson, 2019 [118]	Monitoring socio-economic changes in small towns affected by large scale CSG-LNG development	• Following large-scale CSG development in QLD, regional capitals receive the most economic benefit.<br>• Smaller towns are more affected by these 'booms' (positive or negative).<br>• CSG development increased the number of business and turnover but two years later this was not sustained.

Note: FIFO = fly-in fly-out, DIDO = drive-in drive-out, K10 = Kessler Psychological Distress Scale, SES = socioeconomic status, BMI = body mass index, SF-8 = Short-form 8, EAP = employee assistance program, CPR = cardiopulmonary resuscitation, AED = automated external defibrillator, CCTV = closed circuit television, ECG = electrocardiogram, OH & S =occupational health and safety, GP = general practitioner, PPP = public-private partnership, ED = emergency department, CSG = coal seam gas.

References

1. Alroomi AS, Mohamed S. Occupational stressors and safety behaviour among oil and gas workers in Kuwait: The mediating role of mental health and fatigue. Int J Environ Res Pub Health. 2021;18:21.
2. Bauerle T, Dugdale Z, Poplin G. Mineworker fatigue: A review of what we know and future decisions. Min Eng. 2018;70:33-40.
3. Benson C, Dimopoulos C, Argyropoulos CD, Varianou Mikellidou C, Boustras G. Assessing the common occupational health hazards and their health risks among oil and gas workers. Saf Sci. 2021; doi: 10.1016/j.ssci.2021.105284.
4. Brešić J, Knežević B, Milošević M, Tomljanović T, Golubić R, Mustajbegović J. Stress and work ability in oil industry workers. Arh Hig Rada Toksikol. 2007;58:399-405.
5. Carrington K, McIntosh A. A literature review of wellness, wellbeing and quality of life issues as they impact upon the Australian mining sector. https://eprints.qut.edu.au/66724/ (2013). Accessed 16 Nov 2022.
6. Considine R, Tynan R, James C, Wiggers J, Lewin T, Inder K, et al. The contribution of individual, social and work characteristics to employee mental health in a coal mining industry population. PLOS ONE. 2017; doi:10.1371/journal.pone.0168445.
7. Deng H, He D, Li F. Factors influencing job burnout and musculoskeletal disorders among coal miners in the Xinjiang Uygur Autonomous Region. Pain Res Manag. 2021; doi:10.1155/2021/6629807.
8. Gibson-Smith KL. Promoting and implementing self care: A mixed methods study of offshore workers and remote healthcare practitioners. https://rgu-repository.worktribe.com/output/248874/promoting-and-implementing-self-care-a-mixed-methods-study-of-offshore-workers-and-remote-healthcare-practitioners (2016). Accessed 11 Jan 2023.
9. Gibson Smith K, Paudyal V, Klien S, Stewart D. Health, self-care and the offshore workforce: Opportunities for behaviour change interventions, an epidemiological survey. Rural Remote Health. 2018; doi:10.22605/RRH4319.
10. Hagan-Haynes K, Ramirez-Cardenas A, Wingate KC, Pratt S, Ridl S, Schmick E. On the road again: A cross-sectional survey examining work schedules, commuting time, and driving-related outcomes among U.S. oil and gas extraction workers. Am J Ind Med. 2022; doi:10.1002/ajim.23405.
11. James C, Tynan R, Roach D, Leigh L, Oldmeadow C, Rahman M, et al. Correlates of psychological distress among workers in the mining industry in remote Australia: Evidence from a multi-site cross-sectional survey. PLoS One. 2018; doi: 10.1371/journal.pone.0209377.
12. James CL, Tynan RJ, Bezzina AT, Rahman M, Kelly BJ. Alcohol Consumption in the Australian mining industry: The role of workplace, social, and individual factors. Workplace Health Saf. 2021; doi: 10.1177/21650799211005768.
13. Kvalheim SA, Dahl Ø. Safety compliance and safety climate: A repeated cross-sectional study in the oil and gas industry. J Safety Res. 2016; doi: 10.1016/j.jsr.2016.10.006.
14. Le AB, Balogun AO, Smith TD. Long work hours, overtime, and worker health impairment: A cross-sectional study among stone, sand, and gravel mine workers. Int J Environ Res Pub Health. 2022; doi: 10.3390/ijerph19137740.
15. Liu S, Nkrumah ENK, Akoto LS, Gyabeng E, Nkrumah E. The state of occupational health and safety management frameworks (OHSMF) and occupational injuries and accidents in the Ghanaian oil and gas industry: Assessing the mediating role of safety knowledge. Biomed Res Int. 2020; doi: 10.1155/2020/6354895.
16. McLean KN. Mental health and well-being in resident mine workers: Out of the fly-in fly-out box. Aust J Rur Health. 2012; doi:10.1111/j.1440-1584.2012.01267.x.
17. McPhedran S, De Leo D. Relationship quality, work-family stress, and mental health among Australian male mining industry employees. J Relationsh Res. 2014; doi: 10.1017/jrr.2014.3.
18. Miller P, Brook L, Stomski NJ, Ditchburn G, Morrison P. Suicide risk and social support in Australian resource sector employees: A cross-sectional study. J Community Psychol. 2019; doi: 10.1002/jcop.22145.

19. Miller PB, Brook L, Stomski NJ, Ditchburn G, Morrison P. Depression, suicide risk, and workplace bullying: A comparative study of fly-in, fly-out and residential resource workers in Australia. Aust Health Rev. 2020; doi: 10.1071/AH18155.
20. Moscicka-Teske A, Sadlowska-Wrzesinska J, Najder A, Butlewski M. The relationship between psychosocial risks and occupational functioning among miners. Int J Occup Med Env. 2019; doi: 10.13075/ijomeh.1896.01162.
21. Pavičić Žeželj S, Cvijanović Peloza O, Mika F, Stamenković S, Mahmutović Vranić S, Šabanagić Hajric S. Anxiety and depression symptoms among gas and oil industry workers. Occup Med. 2019; doi: 10.1093/occmed/kqy170.
22. Pelders J, Nelson G. Contributors to fatigue of mine workers in the South African gold and platinum sector. Saf Health Work, 2019; doi:10.1016/j.shaw.2018.12.002.
23. Rasmussen HB, Ahsan D. The safety programme as a tool of improvement for safety culture in the workplace: An exploratory follow-up study from the Danish offshore oil and gas sector. Int J Occup Saf Ergon. 2022; doi:10.1080/10803548.2021.1985303.
24. Roche AM, Lee NK, Battams S, Fischer JA, Cameron J, McEntee A. Alcohol use among workers in male-dominated industries: A systematic review of risk factors. Saf Sci. 2015; doi: 10.1016/j.ssci.2015.04.007..
25. Roche AM, Pidd K, Fischer JA, Lee N, Scarfe A, Kostadinov V. Men, work, and mental health: A systematic review of depression in male-dominated industries and occupations. Saf Health at Work. 2016; doi:10.1016/j.shaw.2016.04.005.
26. Ryan F, Otto B, Khan A, Johnston V. A cross-sectional study of work-related and lifestyle factors associated with the health of Australian long distance commute and residential miners. Eur J Physiother. 2017; doi:10.1080/21679169.2017.1381324.
27. Sinha A, Vyas H. Monitoring health and safety of oilfield workers through wearable technology: SPE International Conference and Exhibition on Health, Safety, Security, Environment, and Social Responsibility. 2018; doi:10.2118/190552-MS.
28. Tynan RJ, Considine R, Wiggers J, Lewin TJ, James C, Inder K, et al. Alcohol consumption in the Australian coal mining industry. Occup Environ Med. 2017; doi:10.1136/oemed-2016-103602.
29. Yeoman K, Sussell A, Retzer K, Poplin G. Health risk factors among miners, oil and gas extraction workers, other manual labor workers, and nonmanual labor workers, BRFSS 2013-2017, 32 States. Workplace Health Saf. 2020; doi:10.1177/2165079920909136.
30. Asare BY, Kwasnicka D, Powell D, Robinson S. Health and well-being of rotation workers in the mining, offshore oil and gas, and construction industry: A systematic review. BMJ Glob Health. 2021; doi: 10.1136/bmjgh-2021-005112.
31. Asare BY, Thomas E, Affandi JS, Schammer M, Harris C, Kwasnicka D, et al. Multiple health-related behaviours among Fly-In Fly-Out workers in the mining industry in Australia: A cross-sectional survey during the COVID-19 pandemic. PLoS One. 2022; doi:10.1371/journal.pone.0275008.
32. Asare BY, Robinson S, Powell D, Kwasnicka. Health and related behaviours of fly-in fly-out workers in the mining industry in Australia: a cross-sectional study. Int Arch Occup Environ Health. 2022; doi:10.1007/s00420-022-01908-x.
33. Barclay M, Harris J, Everinghjam J, Kirsch P, Arend S, Shi S, et al. Factors linked to the well-being of fly-in fly-out (FIFO) workers. https://www.csrm.uq.edu.au/publications/factors-linked-to-the-well-being-of-fly-in-fly-out-fifo-workers (2013). Accessed 9 Jan 2023.
34. Bowers J, Lo J, Miller P, Mawren D, Jones B. Psychological distress in remote mining and construction workers in Australia. Med J Aust. 2018; doi:10.5694/mja17.00950.
35. Crosscare. Challenges for FIFO workers in Australia. https://www.migrantproject.ie/wp-content/uploads/2017/10/Challenges-for-FIFO-workers-Crosscare-Migrant-Project-April-2016.pdf (2016). Accessed 9 Jan 2023.
36. Gardner B, Alfrey K, Vandelanotte C, Rebal AL. Mental health and well-being concerns of fly-in fly-out workers and their partners in Australia: A qualitative study. BMJ Open. 2018; doi:10.1136/bmjopen-2017-019516.
37. Goater S, Goater I, Trivett H, Knowles M, Leveritt M, Lynas D. Health promotion in FIFO and resident mine workforces: A case for a wellness-watch program.

https://www.ruralhealth.org.au/12nrhc/wp-content/uploads/2013/06/Goater-Sarah_Trivett-Hugh_ppr.pdf (2013). Accessed 16 Nov 2022.
38. Harris J. The management of risk factors associated with FIFO workers' mental ill-health. https://www.qmihsconference.org.au/wp-content/uploads/qmihsc-2016-writtenpaper-harris.pdf (2016). Accessed 18 Jan 2023.
39. Joyce SJ, Tomlin M, Somerford PJ, Weeramanthri TS. Health behaviours and outcomes associated with fly-in fly-out and shift workers in Western Australia. Intern Med J. 2013; doi:10.1111/j.1445-5994.2012.02885.x.
40. Korneeva Y, Simonova N. Job stress and working capacity among fly-in-fly-out workers in the oil and gas extraction industries in the Arctic. Int J Env Res Pub Health. 2020; doi:10.3390/ijerph17217759.
41. Labra O, Brouillette C, Gingras-Lacroix G, Cousineau T, Quirion J. The overall health of men who do fly-in fly-out work in the mining sector. Am J Mens Health. 2022; doi:10.1177/1557988322112631.
42. Langdon R, Biggs H, Bevan R. Australian fly-in, fly-out operations: Impacts on communities, safety, workers and their families. Work. 2016; doi:10.3233/WOR-162412.
43. Miller P, Brook L, Stomski N, Ditchburn G, Morrison P. Bullying in Fly-In-Fly-Out employees in the Australian resources sector: A cross-sectional study. PLoS ONE. 2020; doi 10.1371/journal.pone.0229970.
44. Parker S, Fruhen L, Burton C, McQuade S, Loveny J, Griffin M, et al. Impact of FIFO work arrangements on the mental health and wellbeing of FIFO workers. https://research-repository.uwa.edu.au/en/publications/impact-of-fifo-work-arrangements-on-the-mental-health-and-wellbei (2018). Accessed 9 Jan 2023.
45. Queensland Parliament Infrastructure, Planning and Natural Resources Committee. Inquiry into fly-in, fly-out and other long distance commuting work practices in regional Queensland. https://documents.parliament.qld.gov.au/committees/IPNRC/2015/FIFO/02-rpt-009-09Oct2015.pdf (2015). Accessed 9 Jan 2023.
46. Rebar AL, Alfrey K, Gardner B, Vandelanotte C. Health behaviours of Australian fly-in, fly-out workers and partners during on-shift and off-shift days: An ecological momentary assessment study. BMJ open. 2018; doi:10. 1136/ bmjopen- 2018023631.
47. Sustainable Built Environment National Research Centre: Challenges for the FIFO/DIDO workforce: Impacts on health, safety and relationships. https://sbenrc.com.au/app/uploads/2014/09/P2.32_IndustryReport_WEB.pdf (2015). Accessed 9 Jan 2023.
48. Torkington AM, Larkins S, Sen Gipta TS. The psychosocial impacts of fly-in fly-out and drive-in drive-out mining on mining employees: A qualitative study. Aust J Rural Health. 2011; doi: 10.1111/j.1440-1584.2011.01205.x.
49. Turner R, Rubin M. Issues affecting mental health at a fly-in-fly-out mine site: A subjective impact ratings approach. J Appl Soc Psychol. 2022; doi:10.1111/jasp.12913.
50. Western Australia Legislative Assembly. The impact of FIFO work practices on mental health. https://www.parliament.wa.gov.au/Parliament/commit.nsf/(Report+Lookup+by+Com+ID)/2E970A7A4934026448257E67002BF9D1/$file/20150617%20-%20Final%20Report%20w%20signature%20for%20website.pdf (2015). Accessed 9 Jan 2023
51. Adams ME, Lazarsfeld-Jensen A, Francis K. The implications of isolation for remote industrial health workers. Rural Remote Health. 2019; doi:10.22605/RRH5001.
52. Dugdale Z, Eiter B, Chaumont Menendez C, Wong I, Bauerle T. Findings from a systematic review of fatigue interventions: What's (not) being tested in mining and other industrial environments. Am J Ind Med. 2022; doi:10.1002/ajim.23334.
53. International Association of Oil and Gas Producers. Health management in the oil and gas industry: An overview. https://www.ipieca.org/resources/health-management-in-the-oil-and-gas-industry (2019). Accessed 31 May 2023.
54. Ling R, Kelly B, Considine R, Tynan R, Searles A, Doran CM. The economic impact of psychological distress in the Australian Coal Mining Industry. J Occup Environ Med. 2016; doi: 10.1097/JOM.0000000000000714.

55. Mosadeghrad AM, Langroudi HR. Health service delivery to oil companies' employees: A comparative review. Hakim Health Sys Res. 2021;24:76-93.
56. Norman N, Valentine MJ. Remote medicine: A textbook for trainee and established remote healthcare practitioners. World Scientific Publishing; 2020.
57. Tynan RJ, Considine R, Rich JL, Skehan J, Wiggers J, Lewin TJ, et al. Help-seeking for mental health problems by employees in the Australian Mining Industry. BMC Health Serv Res. 2016; doi: 10.1186/s12913-016-1755-1.
58. Abhilash KPP, Sivanandan A. Early management of trauma: The golden hour. Curr Med Issues. 2020; doi:10.4103/cmi.cmi_61_19.
59. Acheampong T, Kemp AG. Health, safety and environmental (HSE) regulation and outcomes in the offshore oil and gas industry: Performance review of trends in the United Kingdom Continental Shelf. Saf Sci. 2022; doi:10.1016/j.ssci.2021.105634.
60. Bonato D, Costa AS, Viterbo LMF, Vidal DG, Dinis MAP. Medical Emergency Resource Classification Instrument (MERCI) in the Oil Industry, Brazil. In: Arezes PM, Baptista JS, Barroso MP, Caneiro P, Cordeiro P, Costa N, et al, editors. Occupational and Environmental Safety and Health II. Heidelberg: Springer International Publishing, 2020. p. 247-254.
61. Croser JL. Trauma care systems in Australia. Injury. 2003; doi: 10.1016/S0020-1383(03)00157-8.
62. Enright CA, Harman CS, Brune JF. Advanced life support in the mining environment. https://www.proceedings.com/30888.html (2016). Accessed 16 Nov 2022.
63. Fatovich DM, Phillips M, Langford SE, Jacobs IG. A comparison of metropolitan vs rural major trauma in Western Australia. Resuscitation. 2021; doi:10.1016/j.resuscitation.2011.02.040.
64. Gibson Smith K, Paudyal V, Klein S, Stewart D. Medical evacuations and work absences in offshore oil and gas industry personnel. SelfCare. 2019;10:105-15.
65. Huzaini ASB, Mohammad R, Othman N, Kadir ZA. Exploring of offshore medical emergency response system challenges in oil and gas environment. J Environ Treat Tech. 2020;8:364-73.
66. Ims, B. Emergency preparedness in Arctic oil and gas exploration. https://ntnuopen.ntnu.no/ntnu-xmlui/bitstream/handle/11250/238425/646721_FULLTEXT01.pdf?sequence=1&isAllowed=y (2013). Accessed 9 Jan 2023.
67. International Association of Oil and Gas Producers. Medical emergency response and Primary Healthcare guideline. https://www.iogp.org/bookstore/product/medical-emergency-response-and-primary-healthcare-guideline/ (2022). Accessed 31 May 2023.
68. Ponsonby W, Mika F, Irons G. Offshore industry: Medical emergency response in the offshore oil and gas industry. Occup Med. 2009; doi:10.1093/occmed/kqp075.
69. Sae-Jia T, Sithisarankul P. medical evacuations among offshore oil and gas industries in the Gulf of Thailand. Int Marit Health. 2020; doi:10.5603/imh.2020.0021.
70. Toner S, Andree Wiltens D, Berg J, Williams H, Klein S, Marshall S, et al. Medical evacuations in the oil and gas industry: A retrospective review with implications for future evacuation and preventative strategies. J Travel Med. 2017; doi:10.1093/jtm/taw095.
71. Asare-Doku W, Rich J, Kelly B, James C. Mental health interventions in the mining industry: A narrative review. Ment Health Rev J. 2020; doi:10.1108/MHRJ-10-2019-0039.
72. Bezzina A, Austin EK, Watson T, Ashton L. James CL. Health and wellness in the Australian coal mining industry: A cross sectional analysis of baseline findings from the RESHAPE workplace wellness program. PLOS ONE. 2021; doi:10.1371/journal.pone.0252802.
73. Campbell K, Burns C. Total Worker Health:Implications for the Occupational Health Nurse. Workplace Health Saf. 2015; doi:10.1177/2165079915576921.
74. Fagade OA, Ajayi PA, Okegbemiro SA, Fadeyi OT. Shifting focus in workplace health care services delivery strategy: The experience of Chevron Nigeria: International Conference on Health, Safety, and Environment in Oil and Gas Exploration and Production. 2008; doi:10.2118/111763-MS.
75. Khanal S, Lloyd B, Rissel C, Portors C, Grunseit A, Indig D, et al. Evaluation of the implementation of Get Healthy at Work, a workplace health promotion program in New South Wales, Australia. Health Promot J Austr. 2016; doi:10.1071/HE16039.

76. Feltner C, Peterson K, Palmieri Weber R, Cluff L, Coker-Schwimmer E, Viswanthan M, et al. The effectiveness of Total worker health interventions: A systematic review for a National Institutes of Health pathways to prevention workshop. Ann Intern Med. 2016; doi: 10.7326/M16-0626.
77. National Institute for Occupational Safety and Health. Fundamentals of total worker health approaches: essential elements for advancing worker safety, health, and well-being. https://www.cdc.gov/niosh/docs/2017-112/pdfs/2017_112.pdf?id=10.26616/NIOSHPUB2017112 (2016). Accessed 13 Jun 2023.
78. Sayers E, Roch J, Rahman MM, Kelly B, James C. Does help seeking behavior change over time following a workplace mental health intervention in the coal mining industry? J Occup Environ Med. 2019; doi:10.1097/jom.0000000000001605.
79. Tynan RJ, James C, Considine R, Skehan J, Gullestrup J, Lewin TJ, et al. Feasibility and acceptability of strategies to address mental health and mental ill-health in the Australian coal mining industry. Int J Ment Health Syst. 2018; doi:10.1186/s13033-018-0245-8.
80. Bouabene A. Providing emergency medical care to offshore oil and gas platforms in the Gulf of Mexico using telemedicine: Asia-Pacific Optical and Wireless Communications. 2002; doi:10.1117/12.480636.
81. Anscombe DL. Healthcare delivery for oil rig workers: Telemedicine plays a vital role. Telemed J E Health. 2010; doi:10.1089/tmj.2010.9957.
82. Berg J, Toner S, Stilz R, Klein S, Williams H, Pearson J, et al. Remote health care: a game changer for the Arctic: OTC Arctic Technology Conference. 2015; doi:10.4043/25549-MS.
83. Boniface KS, Shokoohi H, Smith ER, Scantlebury K. Tele-ultrasound and paramedics: Real-time remote physician guidance of the Focused Assessment With Sonography for Trauma examination. Am J Emerg Med. 2011; doi:10.1016/j.ajem.2009.12.001.
84. Cheskes S, McLeod SL, Nolan M, Snobelen P, Vaillancourt C, Brooks SC, et al. Improving access to automated external defibrillators in rural and remote settings: A drone delivery feasibility study. J Am Heart Associ. 2020; doi:10.1161/JAHA.120.016687.
85. Dehours E, Valle B, Bounes V, Girardi C, Tabarly J, Concina F, et al. User satisfaction with maritime telemedicine. J Telemed Telecare. 2012; doi:10.1258/jtt.2012.110910.
86. Dittrick P. Offshore care on line. Oil Gas J. 2009;107:16.
87. Eadie L, Mulhern J, Regan L, Mort A, Shannon H, Macaden A, et al. Remotely supported prehospital ultrasound: A feasibility study of real-time image transmission and expert guidance to aid diagnosis in remote and rural communities. J Telemed Telecare. 2018; doi: 10.1177/1357633X17731444.
88. Evans K, Lerch S, Boyce TW, Myers OB, Kocher E, Cook LS, et al. An innovative approach to enhancing access to medical screening for miners using a mobile clinic with telemedicine capability. J Health Care Poor Underserved. 2016; doi:10.1353/hpu.2016.0182.
89. Evjemo TE, Reegard K, Fernandes A. Telemedicine in Oil and Gas: Current status and potential improvements. Procedia Manuf. 2015; doi:10.1016/j.promfg.2015.07.274.
90. Hellfritz M, Waschkau A, Steinhäuser J. Quality indicators of telemedical care offshore-a scoping review. BMC Health Serv Res. 2021; doi: 10.1186/s12913-021-07303-5.
91. Islam R, Nohara Y, Rahman J, Sultana N, Ahmed A, Nakashima N. Portable health clinic: An advanced tele-healthcare system for unreached communities. In: Ohno-Machando L, Seroussi B, editors. Studies in Health Technology and Informatics. Amsterdam: IOS Press; 2019. p. 616-619.
92. Latifi R. Telemedicine for trauma and emergency care management. In: Latifi R, Doran CR, Merrel RC, editors. Telemedicine, Telehealth and Telepresence: Principles, Strategies, Applications, and New Directions. Switzerland: Springer; 2020. p. 293-305.
93. Mika F, Croitoru E, Dalida R, Sanctis SD. E-health in an International Oil and Gas Company - Saipem's Experience. Acta Inform Med. 2009;17:155-60.
94. Stilz I, de Carvalho F, Toner S, Berg J. A Prospective Investigation of the Impact of Telemedicine and Telemetry on Global Medical Evacuation Rates. J Occup Environ Med. 2022; doi: 10.1097/jom.0000000000002684.
95. Visionflex. How clinical telehealth helps isolated FIFO work teams. https://www.visionflex.com/clinical-telehealth-helps-fifo-work-

teams/#:~:text=It%20improves%20outcomes%20for%20injured,working%20in%20a%20remote%20location (2022). Accessed 9 Jan 2023.
96. Webster K, Fraser S, Mair F, Ferguson J. A low-cost decision support network for electrocardiograph transmission from oil rigs in the North Sea. J Telemed Telecare. 2008; doi: 10.1258/jtt.2008.003021.
97. Woldaregay AZ, Walderhaug S, Hartvigsen G. Literatures review of telemedicine services in maritime and extreme weather. Int J Integr Care. 2016; doi:10.5334/ijic.2597.
98. Woldaregay AZ, Walderhaug S, Hartvigsen G. Telemedicine services for the Arctic: A systematic review. JMIR Med Inform. 2017; doi:10.2196/medinform.6323.
99. ASMOF Doctors' Union. The Doctors Union calls for an end to public private partnerships. https://www.asmofnsw.org.au/Website/Media_Release_Articles/The_Doctors__Union_calls_for_an_end_to_Public_Private_Partnerships.aspx (2021). Accessed 16 Mar 2023.
100. Basabih M, Prasojo E, Rahayu AYS. Hospital services under public-private partnerships, outcomes and, challenges: A literature review. J Public Health Res. 2022; doi: 10.1177/22799036221115781.
101. Bendigo Health. Public Private Partnership. https://www.bendigohealth.org.au/PublicPrivatePartnership/ (2023). Accessed 16 Mar 2023.
102. Eurodad. History repppeated: Why private public partnerships are not the solution. https://www.eurodad.org/historyrepppeated2 (2022). Accessed 16 Mar 2023.
103. Farquhar E, Moran A, Schmidt D. Mechanisms to achieve a successful rural physiotherapy public–private partnership: A qualitative study. Rural Remote Health. 2020; doi:10.22605/RRH5668.
104. Ganapathy K, Reddy S. Technology enabled remote healthcare in Public Private Partnership mode: A Story from India. In: Latifi R, Doran CR, Merrel RC, editors. Telemedicine, Telehealth and Telepresence: Principles, Strategies, Applications, and New Directions. Switzerland: Springer; 2020. p. 197-233.
105. Hall D. Why public-private partnerships don't work: The many advantages of the public alternative. http://www.world-psi.org/en/publication-why-public-private-partnerships-dont-work (2015). Accessed 16 Mar 2023.
106. Infrastructure Partnerships Australia. A submission on the future directions for Public Private Partnerships. https://infrastructure.org.au/wp-content/uploads/2017/01/PPP.pdf (2013). Accessed 26 May 2023.
107. Institute for Global Health Sciences. PPPs in healthcare: Models, lessons and trends for the future. https://globalhealthsciences.ucsf.edu/sites/globalhealthsciences.ucsf.edu/files/ppp-report-series-business-model.pdf (2018). Accessed 16 Mar 2023.
108. Jefferies M, Gajendran T, Brewer, G. Public private partnerships: The provision of healthcare infrastructure in Australia. https://www.arcom.ac.uk/-docs/proceedings/ar2013-0809-0818_Jefferies_Gajendran_Brewer.pdf (2013). Accessed 16 Mar 2023.
109. Joudyian N, Doshmangir L, Mahdavi M, Tabrizi JS, Gordeev VS. Public-private partnerships in primary health care: a scoping review. BMC Health Serv Res. 2021; doi: 10.1186/s12913-020-05979-9.
110. Makinen M. Working with local communities and health care systems in areas of oil and gas operations: SPE International Conference on Health, Safety and Environment in Oil and Gas Exploration and Production. 2000; doi:10.2118/61516-MS.
111. Tabrizi JS, Azami-Aghdash S, Gharaee H. Public-Private Partnership policy in primary health care: A scoping review. J Prim Care Community Health. 2020; doi:10.1177/2150132720943769.
112. Weinberg A, Laufman L, Parish D, Crossett T. Using the Oil & Gas Producers Guidelines on Strategic Health Management: Achieving sustainable healthcare improvement: SPE International Conference on Health, Safety and Environment in Oil and Gas Exploration and Production. 2002; doi: 10.2118/74037-MS.
113. House of Representatives Standing Committee on Regional Australia. Cancer of the bush or salvation for our cities? https://www.aph.gov.au/parliamentary_business/committees/house_of_representatives_committees?url=ra/fifodido/report.htm (2013). Accessed 27 Jul 2023.

114. Becker KL. Help wanted: Health care workers and mental health services. An analysis of six years of community concerns from North Dakota's oil boom residents. J Rural Stud. 2018; doi: 10.1016/j.jrurstud.2018.08.012.
115. Constantine S, Battye K. Mining towns-does the boom mean bust for health services: 12th National Rural Health Conference. https://www.ruralhealth.org.au/12nrhc/wp-content/uploads/2013/06/Constantine-Sarah_ppr.pdf (2015). Accessed 21 Nov 2022.
116. Rifkin W, Witt K, Everingham J, Uhlmann V. Benefits and burdens for rural towns from Queensland's onshore gas development: SPE Asia Pacific Unconventional Resources Conference and Exhibition. 2015; doi:10.2118/176941-MS.
117. Sigal ME. Socioeconomic effects of oil drilling: The case of Ecuador. https://economics.yale.edu/sites/default/files/2023-01/Eliscovich%20Sigal%20Senior%20Essay2016.pdf (2016). Accessed 11 Jan 2023.
118. Witt K, Simpson J. Monitoring socio-economic changes in small towns affected by large scale CSG-LNG development: SPE/AAPG/SEG Asia Pacific Unconventional Resources Technology Conference. 2019; doi:10.15530/AP-URTEC-2019-198314.
